# Tregs dysfunction aggravates postoperative cognitive impairment in aged mice

**DOI:** 10.1186/s12974-023-02760-7

**Published:** 2023-03-17

**Authors:** Yile Zhou, Huihui Ju, Yan Hu, Tingting Li, Zhouyi Chen, Yuan Si, Xia Sun, Yi Shi, Hao Fang

**Affiliations:** 1 Department of Anesthesiology, Zhongshan Hospital, Fudan University, Shanghai, China; 2 Institute of Clinical Science, Zhongshan Hospital, Fudan University, Shanghai, China; 3 Shanghai Key Laboratory of Organ Transplantation, Zhongshan Hospital, Fudan University, Shanghai, China; 4 Department of Anesthesiology, Minhang Branch, Zhongshan Hospital, Fudan University, Shanghai, China; 5 Department of Anesthesiology, Shanghai Cancer Center, Fudan University, Shanghai, China

**Keywords:** Postoperative cognitive dysfunction, Regulatory T cells, Aging, Neuroinflammation, Blood–brain barrier

## Abstract

**Objectives:**

Enhanced neuroinflammation is an important mechanism underlying perioperative neurocognitive disorders. Regulatory T cells (Tregs) play a crucial role in regulating systemic immune responses. The present study was aimed to investigate the participation of Tregs in the development of postoperative cognitive dysfunction (POCD).

**Methods:**

Surgery-associated neurocognitive disorder was induced in 18-month-old mice subjected to internal fixation of tibial fracture. Morris water maze was used to examine mice cognitive function. Splenic Tregs were collected for RNA sequencing and flow cytometry. Levels of inflammatory factors in the circulation and hippocampus were measured by enzyme-linked immunosorbent assay. Protein presences of tight junction proteins were detected by immunofluorescence.

**Results:**

Surgery of internal fixation of tibial fracture induced cognitive impairment in aged mice, accompanied by elevated plasma levels of inflammatory factors and increased circulating Tregs. Transfusion of Tregs from young mice partially restored the structure of the blood–brain barrier and alleviated POCD in aged mice. Compared with young Tregs, differentially expressed genes in aged Tregs were enriched in tumor necrosis factor (TNF) signaling pathway and cytokine–cytokine receptor interaction. Flow cytometry revealed that aged Tregs had blunted functions under basal and stimulated conditions. Blockade of the CD25 epitope protected the blood–brain barrier structure, reduced TNF-α levels in the hippocampus, and improved surgery-associated cognition in aged mice.

**Conclusions:**

Blocking peripheral regulatory T cells improves surgery-induced cognitive function in aged mice. Therefore, aged Tregs play an essential role in the occurrence of POCD.

**Supplementary Information:**

The online version contains supplementary material available at 10.1186/s12974-023-02760-7.

## Introduction

Postoperative cognitive dysfunction (POCD) is presented with impaired learning capacity, memory loss, confusion, anxiety, and personality changes. It is reported that 25.8% of patients exhibit some cognitive disorder in the 1st week after surgeries, of which 9.9% of senile patients remain cognitive impairment longer than 3 months [1]. Aging is a critical risk factor for the occurrence of POCD, as suggested by its features of cognitive impairment which are comparable to those in disease conditions, including neurodegenerative disease [2, 3].

The blood–brain barrier (BBB) provides primary protection for the central nervous system (CNS) against pathological stimuli [4]. Tight junction proteins in vascular endothelial cells of the BBB, including claudin1 and claudin5, is crucial for preventing harmful solutes from passing to the central nervous system [5]. Reduced expressions of junction proteins alter the permeability of the blood–brain barrier, resulting in overspills of plasma proteins and invasion of peripheral cells as well as pathogens [2, 6, 7]. The presence of lymphocytes in the brains of Alzheimer’s patients [8] and aged mice [9] is associated with a disruption of the blood–brain barriers, leading to sterile neuroinflammation in the central nervous system. Of importance, elevated levels of interleukin-6 (IL-6), C-reactive protein, and chitinase 3-like protein in cerebrospinal fluid of aged patients [10] are positively correlated with their cognitive disorders [8], suggesting that chronic neuroinflammation takes part in cognitive impairment [11–13].

Regulatory T cells (Tregs), characterized by high expressions of cluster of differentiation 4 (CD4), CD25 (also known as interleukin 2 receptor alpha), and forkhead box protein P3 (Foxp3), play an obligatory role in immune homeostasis by suppressing excessive immune responses [14]. However, the role of Tregs in the central nervous system is inconclusive [15, 16]. In Alzheimer’s mice, transient depletion of Tregs promotes β-amyloid plaque clearance by inducing leukocytes recruitment through the choroid plexus [17], but accelerates memory loss by limiting the recruitment of microglia toward amyloid plaques [18]. It is also reported that Tregs inhibit astrogliosis and promote neural recovery [19], but impair cerebral microvasculature [20] in a mouse stroke model.

Thus, the present study was designed to investigate the participation of Tregs in a mouse POCD model subjected to tibial fractures internal fixation surgery.

## Materials and methods

### Animals

Male C57BL/6 mice, 18-month-old and 10-week-old, were purchased from Shanghai Jiesijie Company (Shanghai, China). B6.129(Cg)-Foxp3^tm4(YFP/icre)Ayr^/J (Foxp3^YFP^) transgenic mice were used on the C57BL/6 background (Cyagen, China). Mice had free access to food and water. All animals were housed separately under specific pathogen-free conditions with 12-h light/dark cycles in the Laboratory Animal Unit of Zhongshan Hospital, Fudan University (Shanghai, China). The experimental design was approved by the Animal Ethics Committee of Zhongshan Hospital, Fudan University [SYXK (Shanghai) 2021-#0022].

### POCD model and Tregs intervention

The POCD model was built by conducting internal fixation of tibial fractures as previously described [13]. Briefly, mice were anesthetized with 1% sodium pentobarbital (8 mg/kg, intraperitoneal injection). A 0.3–0.6 cm vertical incision was made near the tibial tubercle, followed by a needle insertion into the tibial tubercle. After the surgery, butorphanol (2 mg/kg) was administered subcutaneously to relieve the pain.

**Fig. 1:**
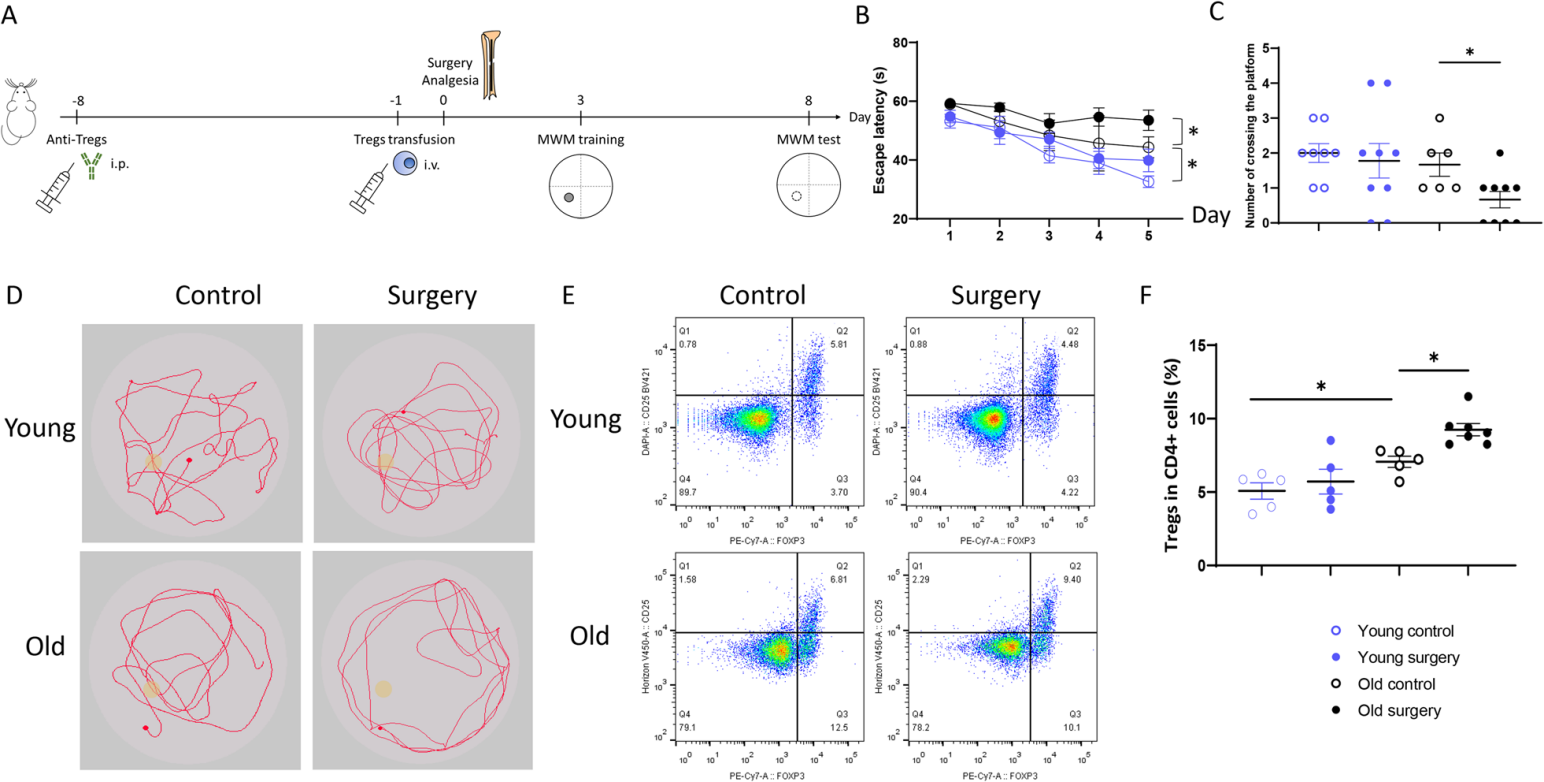
Aged mice have impaired cognitive function after surgery. **A** Experimental scheme of the research. **B** Escape latency of young and old mice after surgery. **C** Number of crossing the target platform of young and old mice after surgery. *P < 0.05 *n* = 6–9. **D** Representative tracing in the swimming pool on the test day. Yellow blocks in the pool indicated the target platform in the acquisition training. **E**, **F**. Number of peripheral Tregs in young and old mice after surgery. *P < 0.05 *n* = 5–7

To block Tregs function, an anti-CD25 antibody (500 µg/mouse; 553864, BD Pharmingen, USA) and its isotopic antibody were administered intraperitoneally [21, 22] (Fig. 1A).

To increase peripheral Tregs, all-trans-retinoic acid (ATRA, 8 mg/kg for young mice, 4 mg/kg for aged mice; R2625, Sigma, Germany) was injected intraperitoneally every 48 h for a week. [23] Four injections of ATRA significantly increased counts of splenic CD4+CD25+Foxp3+ Tregs, but not CD4 or CD8 cells in young mice. However, ATRA had lethal effects on aged mice (Additional file 1: Fig. S1A–C). Therefore, transfusion of Tregs was used to increase peripheral Tregs in the present study. Splenic Tregs from young or aged mice were isolated by a regulatory T cell isolation kit (130-091-041, Miltenyi Biotec, Germany) and verified by flow cytometry (Additional file 1: Fig. S2). A total of 2 × 10^6^ Tregs were intravenously injected through the tail vein 1 day before the surgery [24]. Aged mice were transfused with Tregs from young or aged mice (Fig. 1A).

### Morris water maze

The hippocampus-dependent spatial learning and memory capacity were examined by the Morris water maze test [2, 25, 26]. In brief, mice were acclimated to the maze for 3 days. Spatial acquisition training was conducted for 5 consecutive days (D3–D7). In the Morris test (D8), swimming paths, counts of the target platform crossing, and time spent on each quadrant were documented (Jiliang, China) (Fig. 1A).

### Flow cytometry

To obtain Tregs from spleens, spleens were carefully collected after mice were anesthetized. The spleens were grounded and filtered through a 70 µm filter for single-cell suspensions. Tregs were incubated with surface antibodies and viability stain (565388, BD Pharmingen, USA) for 30 min at 4 °C. After permeabilization with Foxp3/Transcription Factor Staining Buffer (00-5523-00, Thermo Fisher, USA), the cells were further incubated with intracellular antibodies for 30 min at 4 °C (Table 1). A separate group of cells was challenged with a leukocyte activation cocktail (0.2 µl/10^6^ cells; 550583, BD Pharmingen, USA) containing phorbol 12-myristate-13-acetate (PMA), ionomycin, and brefeldin-A for 4 h, following the protocol of the manufactory. The flow cytometry assays were performed on a BD FAC Symphony (BD Germany).

**Table 1:** Antibody for flow cytometry

Panel 1		Panel 2	
Antibody	Product	Antibody	Product
Fixable viability stain	BD Pharmingen-565388	Fixable Viability Stain	BD Pharmingen-565388
CD4	BD Pharmingen-563106	CD4	BD Pharmingen-563106
CD25	Biolegend-102033	CD25	Biolegend-102033
FOXP3	eBioscience-25-5773-80	FOXP3	eBioscience-25-5773-80
CCR4	Biolegend-131219	5’-NT	Biolegend-127205
CTLA-4	BD Pharmingen-565778	Granzyme B	Biolegend-372215
IL-2	Biolegend-503829	Helios	Biolegend-137204
IRF-4	BD Pharmingen-566649	IL-10	Biolegend-505031
LAG-3	BD Pharmingen-740959	Perforin-1	Biolegend-154303
LRRC32	Biolegend-142905	TNFRSF4	BD Pharmingen-740545
NTPDase 1	Biolegend-143811	TNFRSF18	BD Pharmingen-741020
PD-1	Biolegend-135213	TGF-β1	Biolegend-141409

### Enzyme-linked immunosorbent assay (ELISA)

Mice hippocampal tissue (10 mg) or plasma (100 µl) were collected for ELISA. Levels of cytokines in plasma and hippocampal tissues were measured using Bioplex suspension chip reagent Bio-Plex Pro Mouse Cytokine 23-plex (M60009RDPD, Biorad, USA) and normalized with protein concentration in samples.

### Gene-expression profiling assay

Gene-expression profiling assays on splenic Tregs, collected from both young and aged mice, were performed by the Shanghai Institute of Immunology. The gene expression files were analyzed with R-3.4.1 software. Differentially expressed genes (DEGs) were defined when an adjusted P value was less than 0.05. DEGs were calculated with the limma package [27]. Database for Annotation, Visualization and Integrated Discovery (v6.8) was used to analyze gene function and potential pathways [28]. Bubble Plots were performed by the ggplot2 package [29]. Ligand–receptor interaction analysis was performed using the iTALK package [30].

### Immunofluorescence

To examine the blood–brain barrier permeability, 40 kDa dextran (20–25 mg/kg; D1829 Thermo Fisher, USA) was injected via the tail vein 24 h before the euthanization. After anesthetizing with pentobarbital, the mice were transcardially perfused with 40 ml ice-cold PBS for 20 min. Brain samples were dehydrated in the 30% (w/v) sucrose solution and embedded in an optimum cutting temperature compound (OCT, 4583, Sakura, USA). The frozen brain tissue was prepared in 5-μm thickness. Brain slides were blocked with 5% goat serum and then incubated with primary antibodies, anti-CD31 (24590, Abcam, UK), anti-claudin1 (15098, Abcam, UK), and anti-claudin5 (15106, Abcam, UK), overnight at 4 °C. On the 2nd day, slides were incubated with secondary antibodies for 1 h at 37 °C. Nuclei were stained with 4′,6-diamidino-2-phenylindole (DAPI) for 10 min at 37 °C. Images were taken using a fluorescence microscope (Olympus BX51, Japan).

The presence of Tregs in the hippocampus was examined by immunofluorescence in two approaches. Tregs from Foxp3^YFP^ mice, in which the Foxp3 protein was knocked in a yellow fluorescent protein, were injected into aged mice subjected to the surgery. The YFP signal was detected in the choroid plexus, but not the hippocampi of the mice. (Fig. 1A, Additional file 1: Fig. S3). In addition, brain slides were also incubated with primary antibodies, anti-CD4 (557307, BD, USA) and anti-Foxp3 (NB100-39002SS, Novus, USA).

### Statistical analysis

Prism 9 (GraphPad, USA) software was used in the present study. Data are presented as means ± SEM. Flow cytometry data were analyzed with FlowJo v10.0.8 (BD, USA). The statistical analysis was done by one-way ANOVA followed by post hoc Bonferroni comparison. The comparison between Tregs with or without PMA/Ionomycin stimulation was performed by paired *t* test. *P* < 0.05 was considered statistically significant.

## Results

### Aged mice subjected to the surgery exhibit cognitive dysfunction

In the acquisition course, surgery significantly increased escape latency in aged mice, but not young mice (Fig. 1B). In the maze test, the surgery did not affect cognitive scores in young mice, but significantly reduced target platform crossings in aged mice compared with their age-matched counterparts (Fig. 1C, D).

In aged mice, but not young ones, the surgery of internal fixation of tibial fractures significantly increased splenic Tregs, and the increase occurred since day 1 (Fig. 1E, F).

**Fig. 2:**
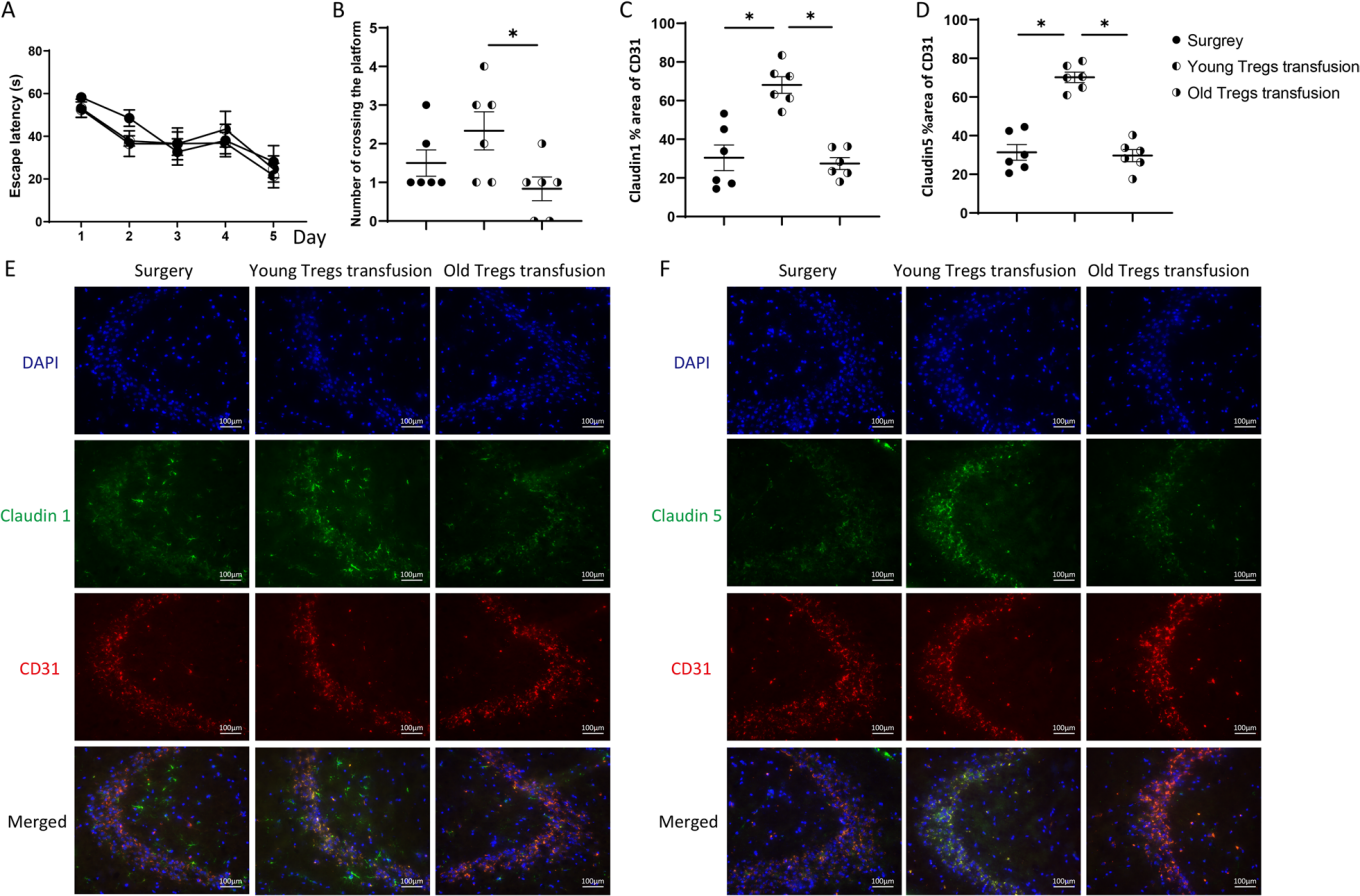
Tregs transfusion changes mice cognitive function and structure of the blood–brain barrier. **A** Escape latency of old mice with Tregs transfusion. **B** Counts of crossing the target platform of old mice with Tregs transfusion. *P < 0.05 *n* = 6. Quantification of claudin1 (**C**) and claudin5 (**D**) in the CA3 region of the hippocampus. Representative immunofluorescence signals of claudin1 (**E**) and claudin5 (**F**). DAPI labeled the nuclei (blue), CD31 labeled endothelial cells (red), and claudin1 or claudin5 stained green. Magnification, ×200, *P < 0.05 *n* = 6

In the maze test, aged mice transfused with young Tregs had more crossings on the target platform than those transfused with aged Tregs (Fig. 2A, B). Transfusion with young Tregs, but not aged Tregs, increased protein presence of junction protein claudin1 and claudin5 in the CA3 region of the hippocampus of aged mice (Fig. 2C-F). Transfused Tregs were not detected in the hippocampi of aged mice (Additional file 1: Fig. S3). In addition, transfusion with young Tregs accelerated the swimming speed of aged mice (Additional file 1: Fig. S4).

Of note, ARTA administration, a pharmacological approach to increase peripheral Tregs, had lethal effects on aged mice (Additional file 1: Fig. S1).

### Aged mice possess impaired Tregs

To explore Tregs functions in aging, splenic Tregs of young and aged mice were collected for RNA-sequencing and flow cytometry.

**Fig. 3:**
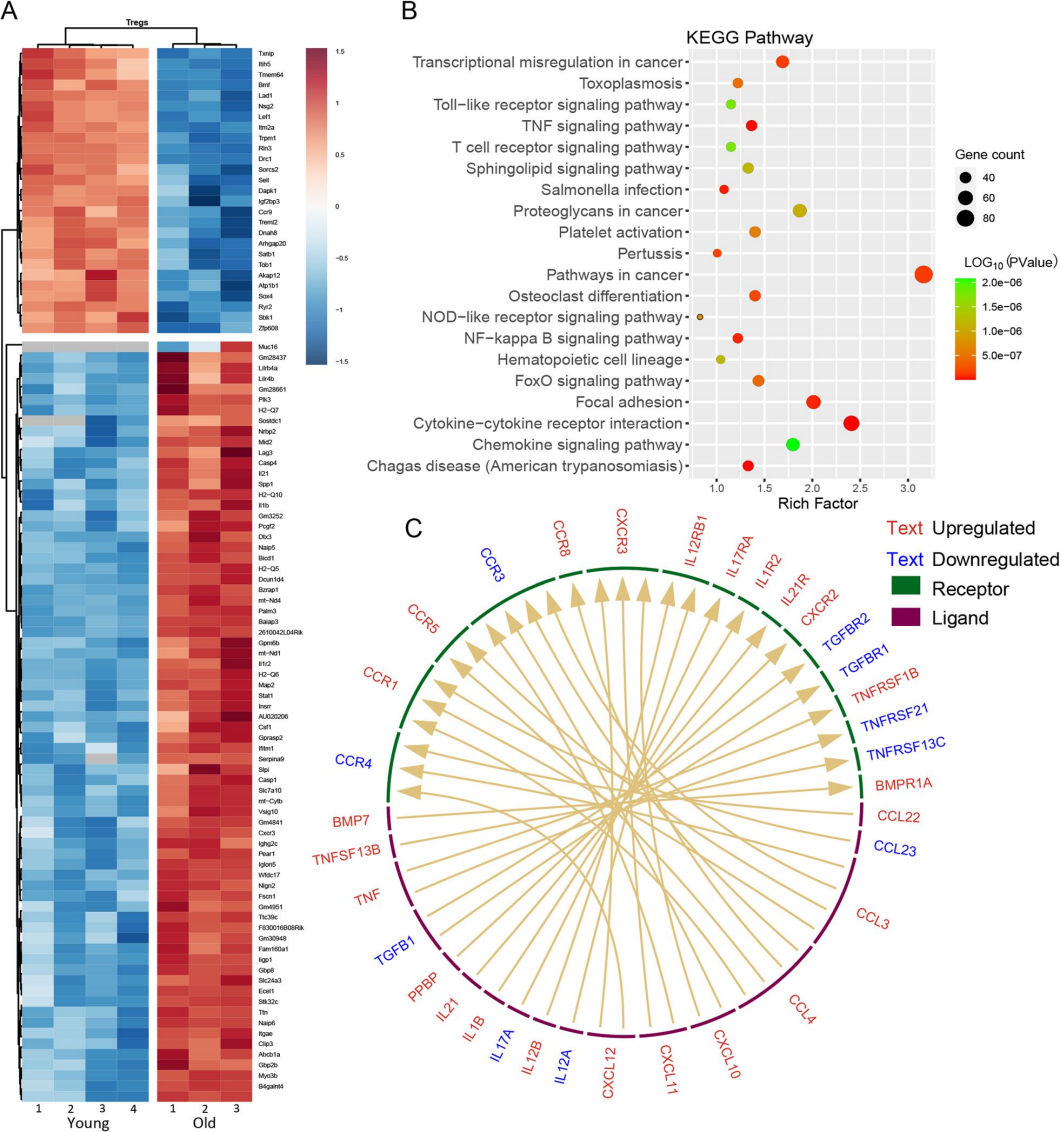
Aging changes Tregs function. **A** Heatmap of top 100 DEGs in splenic Tregs. **B** Top 20 pathways of DEGs in KEGG analysis. **C** Cytokine–cytokine receptor interactions prediction network with DEGs. Upregulated genes in red and downregulated genes in blue. Arrow links ligand and its pertinent receptors

A total of 2910 DEGs were identified in Tregs of young and aged mice (Fig. 3A). Kyoto Encyclopedia of Genes and Genomes pathway analysis revealed that DEGs were enriched in cytokine–cytokine receptor interaction, Chagas disease, tumor necrosis factor (TNF) signaling pathway, Salmonella infection, and NF-kappa B signaling pathway (Fig. 3B). Ligand–receptor interaction analysis confirmed that TNF was paired with upregulated tumor necrosis factor receptor superfamily member (TNFRSF) 1B and downregulated TNFRSF21, while upregulated TNFSF13B was paired with downregulated TNFRSF13C. In addition, increased expressions of transforming growth factor-β1 (TGF-β1) corresponded with downregulated TGF-β1 receptors 1 and 2 (TFGBR1 and TGFBR2). Interleukin (IL)-1β, IL-12, and IL-17 were paired with IL-1 receptor2 (IL-1R2), IL-12 receptor B1 (IL12B1), and IL-17 receptor (IL17RA), respectively. C–C motif ligand (CCL) 3 was paired with downregulated C–C motif chemokine receptor (CCR) 3 and CCR4, as well as upregulated CCR1 and CCR5. CCL4 was paired with downregulated CCR4 and upregulated CCR1, CCR5, and CCR8 (Fig. 3C).

**Fig. 4:**
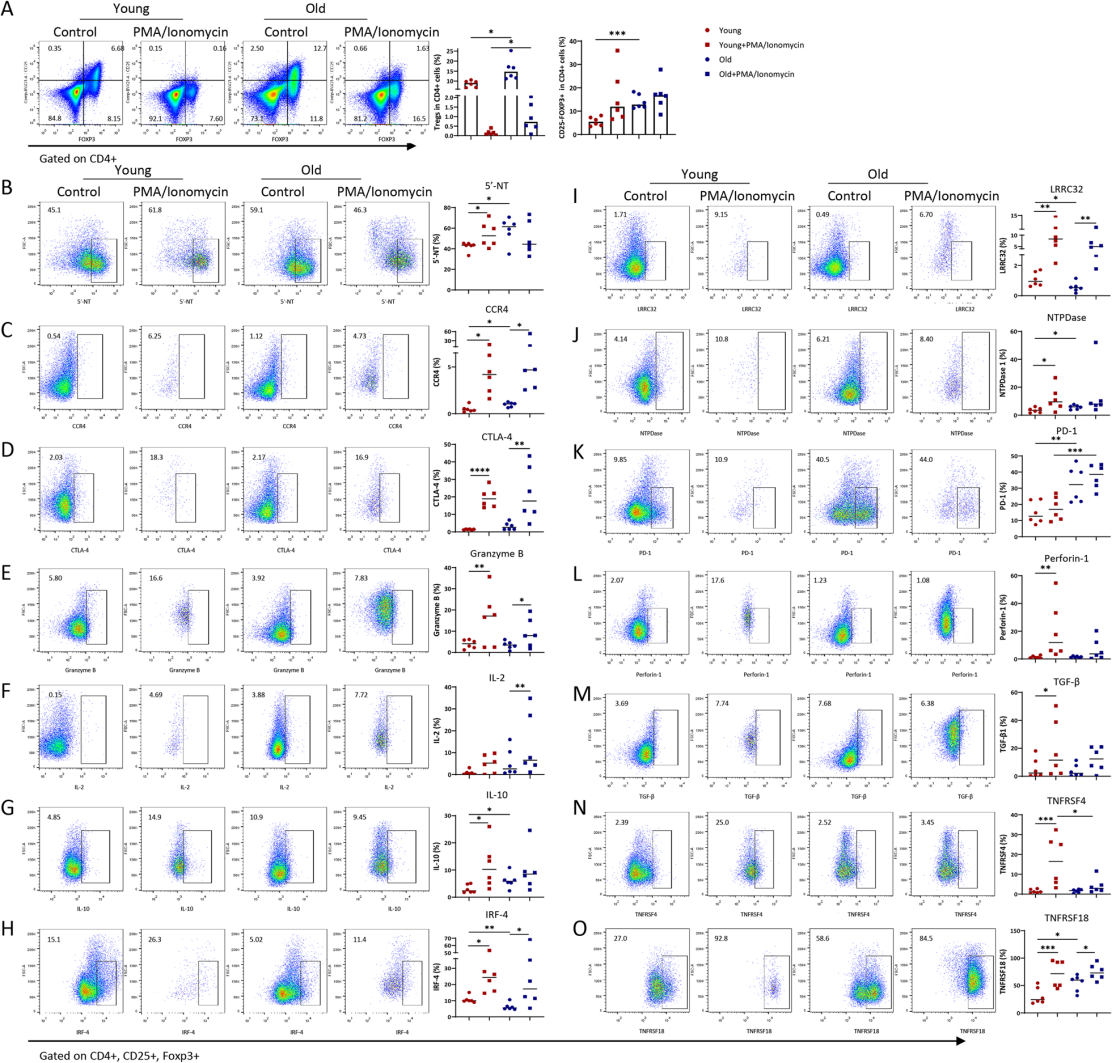
Changes of candidate proteins in Tregs under basal and stimulated conditions. **A** Tregs and CD25-Foxp3 cells proportion in CD4^+^ cells. Changes of candidate proteins **B** 5-NT, **C** CCR4, **D** CTLA-4, **E** GranzymeB, **F** IL-2, **G** IL-10, **H** IRF-4, **I** LRRC32, **J** NTPDase, **K** PD-1, **L** Perforin-1, **M** TGF-β, **N** TNFRSF4, and **O** TNFRSF18 in Tregs under basal and stimulated conditions. *P < 0.05 *n* = 6

Flow cytometry revealed that aged mice had higher counts of CD4+CD25+Foxp3+ Tregs and CD4+CD25-Foxp3+ cells than their young counterparts. Under the basal condition, compared with young mice, aged mice had increased protein expressions of 5′-nucleotidase (5′-NT), CCR4, IL-10, ectonucleoside triphosphate diphosphohydrolase 1 (NTPDase 1), programmed cell death protein 1 (PD-1), and TNFRSF18, reduced expressions of interferon regulatory factor-4 (IRF-4) and leucine-rich repeat-containing 32 (LRRC32), and unchanged expressions of cytotoxic T-lymphocyte protein 4 (CTLA-4), IL-2, zinc finger protein Helios, lymphocyte activation gene 3 protein (LAG-3), and TNFRSF4. Combined stimulation of PMA and ionomycin induced comparable expressions of CCR4, CTLA-4, Granzyme B, IRF-4, LRRC32, and TNFRSF18 in Tregs of both young and old mice. The stimulation did not increase 5′-NT, NTPDase 1, IL-10, Perforin-1, TGF-β1, or TNFRSF4 protein expressions in the aged Tregs. The simulation significantly increased IL-2 expression in aged, but not in young, ones (Fig. 4, Additional file 1: Fig. S5).

### Blocking the CD25 molecule improves cognitive function in aged mice subjected to surgery

Taken together with data on Tregs dysfunction in aged mice and their impaired cognitive performance, it is plausible that Tregs play a role in the occurrence of POCD in aged mice. To further study the participation of Tregs in POCD, the anti-CD25 antibody was applied in aged mice [31, 32]. Blockade of CD25 did not affect the body weight or general condition in aged mice (Additional file 1: Fig. S6).

**Fig. 5:**
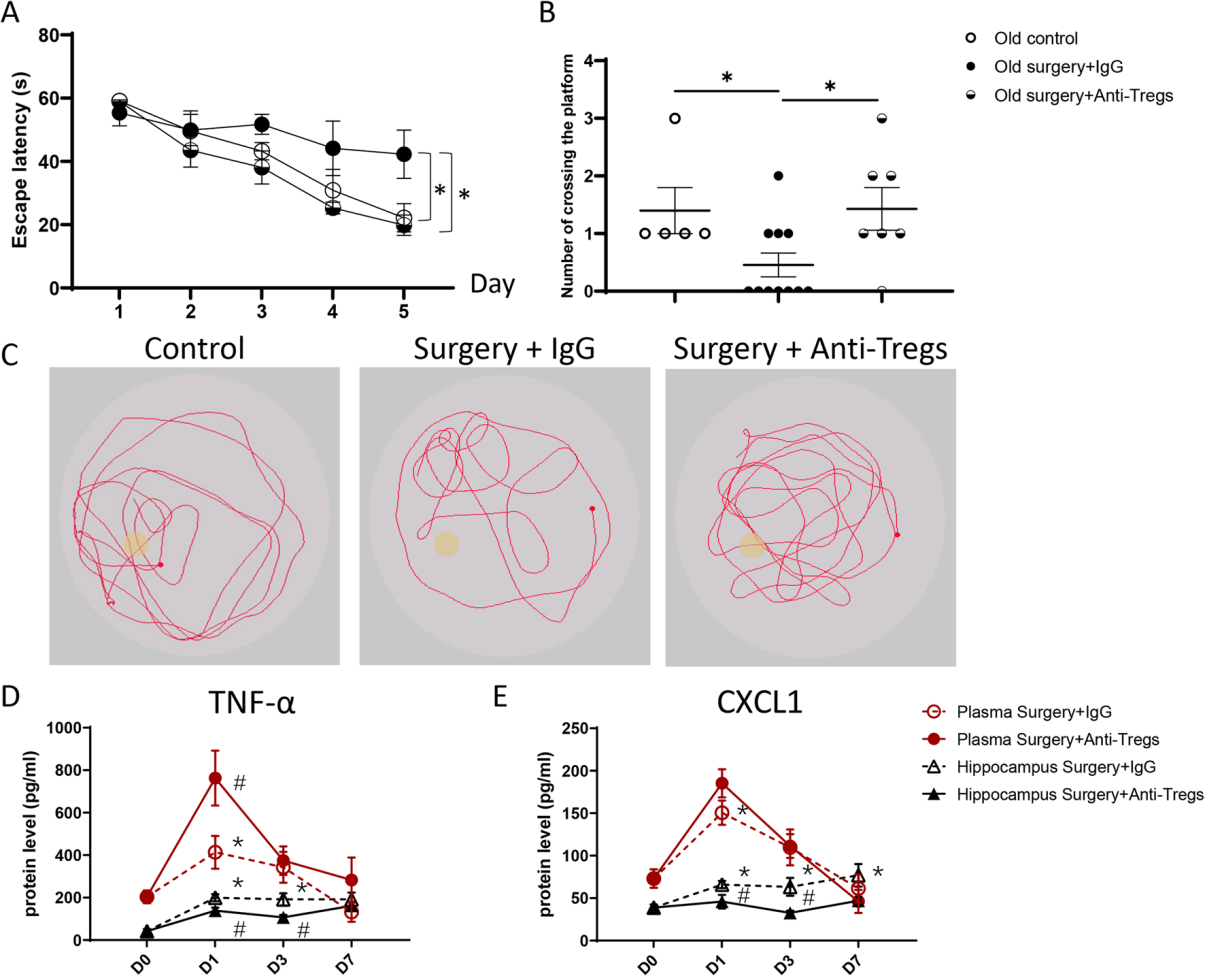
Tregs ablation restores POCD in aged mice. **A** Escape latency in mice. **B** Number of crossing the target platform. **C** Representative tracing in the swimming pool on the test day (D8). **D** Protein expression of TNF-α of mice plasma and hippocampi after the surgery. **E** Protein expression of CXCL1 of mice plasma and hippocampi after the surgery *P < 0.05 vs. D0, ^#^*P* < 0.05 vs. Surgery + IgG on the same day. *n* = 5–11

Blocking CD25 reduced the escape latency in aged mice compared with those administered with the isotopic antibody (Fig. 5A). In the Morris test, administration with the anti-CD25 antibody significantly increased crossing counts in the target platform than those with the isotopic antibody (Fig. 5B, C).

The surgery transiently increased plasma levels of IL-1β, IL-6, IL-10, TNF-α, granulocyte–macrophage colony-stimulating factor (GM-CSF), interferon-γ (IFN-γ), CCL2 and C–X–C motif chemokine 1 (CXCL1) expression on day 1. Blockade of the CD25 epitope increased plasma TNF-α level when compared with the isotopic antibody, but did not affect plasma levels of other inflammatory factors (Fig. 5D, E. Additional file 1: Fig. S7).

In mouse hippocampus, the surgery significantly and consistently increased IL-3, IL-4, IL-5, IL-6, IL-10, IL-12, IL-17, TNF-α, GM-CSF, IFN-γ, CCL2, CCL3 and CXCL1 levels. Blockade of the CD25 epitope partially reduced IL-3, TNF-α, and CXCL1 levels in the hippocampus of aged mice but did not affect levels of other inflammatory factors (Fig. 5D, E, Additional file 1: Fig. S7).

Fluorescent signals of Tregs, CD4, CD25, or Foxp3, were not detected in the brain in young or aged mice subjected to the surgery (Additional file 1: Fig. S3).

**Fig. 6:**
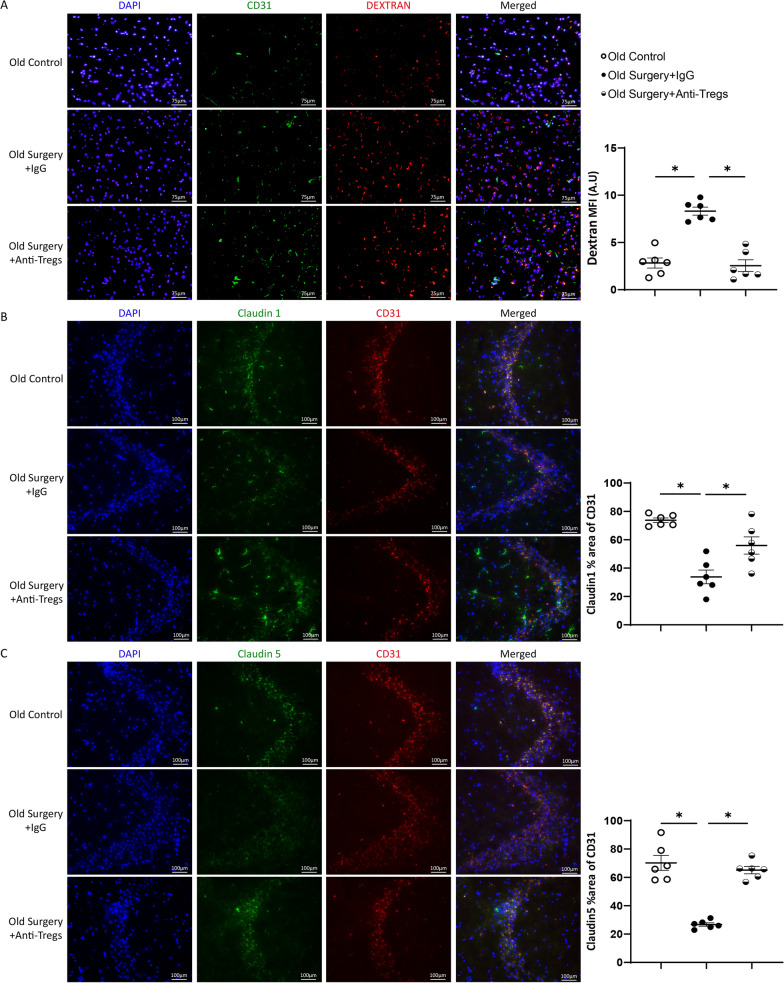
Tregs ablation restores the structure of the blood–brain barrier in aged mice. **A** Fluorescence signals and quantification of 40 kDa dextran in the mouse hippocampus. DAPI labeled the nuclei (blue), CD31 labeled endothelial cells (green), and dextran stained red (Texas red). Immunofluorescence signals and quantification of claudin1 (**B**) and claudin5 (**C**) in the CA3 region of the hippocampus. DAPI labeled the nuclei (blue), CD31 labeled endothelial cells (red), claudin1 or claudin5 stained green. Magnification, ×200, *P < 0.05 *n* = 6

BBB permeability was evaluated by fluorescent signals of low-molecule dextran in hippocampi. The signal of low-molecular dextran was observed in the hippocampus of aged mice subjected to the surgery. Compared with isotopic antibody administration, CD25 blockade significantly reduced the fluorescent signals (Fig. 6A). The junction protein, claudin1 and claudin5, were co-localized with CD31+ endothelial cells in the CA3 region of the hippocampus. The surgery significantly reduced the protein presence of junction proteins in mice administrated with the isotype antibody. Compared to the isotope antibody group, CD25 blockade significantly increased the protein presence of claudin1 and claudin5 (Fig. 6B, C).

## Discussion

The present study reports that aging deteriorates Tregs function and impacts mice cognition when subjected to the surgery of internal fixation of tibial fractures. The surgery-associated cognitive impairment in aged mice contributes to the disruption of the blood–brain barrier and enhanced sterile inflammation in the central nervous system. Blocking the CD25 epitope protects the blood–brain barrier, downregulates inflammation in the hippocampus, and restores cognitive function.

Taken into consideration that aged mice had worse cognition than young mice [2, 25], the present study reported that surgery further deteriorates cognitive function in aged mice, supporting the notion that surgery, as an exogenous stimulus, is a potential risk of cognitive dysfunction in the geriatric population [33, 34]. The involvement of Tregs in the development of cognitive impairment was first reported in 2006 [35]. Tregs function was modified by aging, as shown by the abundant differently expressed genes which have extensive involvement in biological processes and the altered responses to stimuli in flow cytometry examination. Together with increased counts of peripheral Tregs upon the surgery, the findings imply that aged Tregs participate in the development of cognitive dysfunction [36, 37]. Indeed, transfusion with young Tregs partially restores cognition in aged mice. Noted, blocking the CD25 epitope [38], the characteristic marker of Tregs, improved cognitive function in aged mice. Thus, the present study provides substantial evidence that aged Tregs are critical to cognitive dysfunction.

Based on the DEGs of aged Tregs and the associated dysfunction from FACS study, aged Tregs are detrimental, especially challenged under pathological conditions. Tregs-mediated immune suppression includes cytolysis, metabolic disruption, and secretion of anti-inflammatory cytokines [39]. Both NTPDase 1 and 5′-NT are cell-surface proteins, in which NTPDase 1 hydrolyzes extracellular ATP and ADP to AMP [40], and 5′-NT converts AMP to anti-inflammatory adenosine [41]. Although aged Tregs had compensatory higher expressions of NTPDase 1 and 5′-NT under the basal condition, the nonresponsiveness of these two molecules in the stimulated state reinforces the incompetence of aged Tregs [41–43].

Tregs participate in cytolysis through Granzyme B and Perforin-1. In the present study, aged Tregs did not increase Perforin-1 expression in response to the stimuli, although Granzyme B expressions were comparable to that of young Tregs, suggesting that the cytotoxic effects of aged Tregs are also blunted [39].

Aged Tregs did not produce stimulated TGF-β1 [44], despite its key regulator LRRC32 was upregulated [45]. Together with the unresponsiveness of IL-10 in aged mice, the present results confirmed the impairment of Tregs in aged mice [46].

Both CTLA-4 and PD-1 are immune checkpoint proteins. Loss of CTLA-4 results in massive lymphocyte proliferation [47]. Increased PD-1 expression exhausts T-cell function [48, 49]. Aged Tregs had increased expression of PD-1 under both basal and stimulated conditions, implying the detrimental role of aged Tregs.

In addition, IL-2 production was higher in stimulated aged Tregs, suggesting that aged Tregs possess pro-inflammatory features. In line, aged Tregs had reduced basal expressions of IRF-4 and increased stimulated CCR4 expression in flow cytometry examination. CCR4 is a critical receptor for chemokines, and IRF-4 is associated with enhanced immunosuppression and differentiation of effector Tregs [50] via regulating IL-17, IL-21 [51], and IL-4 [52] production.

The strategy of CD25 blockade has been used to investigate the role of Treg in renal ischemia–reperfusion injury [53], pancreatic intraepithelial neoplasms [38], and mesothelioma model [54]. In the present study, blocking CD25 increased the plasma TNF-α level in aged mice subjected to the surgery, confirming the immunosuppressive role of Tregs in the circulating immune system. Three TNF receptor superfamily members were identified in the RNA sequencing analyses, including TNFRSF1B, TNFRSF21, and TNFRSF13C. TNFRSF1B, also known as TNFR2, is strictly expressed in neurons, oligodendrocytes, myeloid-derived suppressor cells, Tregs, and monocytes [55–59]. TNFRSF1B mediates Tregs proliferation and function [60, 61]. Thus, the upregulated expression of TNFRSF1B exacerbates inflammatory responses [62–64]. TNFRSF21 is involved in regulating T helper cells, while TNFRSF13C regulates B cells. Furthermore, the increased basal expression of TNFRSF18 and nonresponsiveness of TNFRSF4 in aged Tregs in flow cytometry study indicate that the immunosuppressive effects of Tregs on inflammatory responses are compromised, since TNFRSF18 [65] and TNFRSF4 [66] are responsible for the Tregs suppression and differentiation [67].

Indeed, the surgery transiently increased plasma levels of inflammatory factors, supporting that the surgery per se is an inflammatory stimulus for patients. However, hippocampal levels of TNF-α, CXCL1, IL-1, IL-3, IL-4, IL-5, IL-6, IL-10, IL-12, and IL-17 were maintained at higher levels, indicating that the immune status in the CNS is not synchronized. The increased levels of cytokines in the hippocampus confirmed that enhanced inflammation is a crucial player in POCD. More important, the protracted inflammatory responses in the hippocampus partially explain the prolonged cognitive impairment in senile patients subjected to surgeries. The presence of Tregs in the central nervous system has been reported in mice after ischemic stroke [19] and in mice with experimental autoimmune encephalomyelitis [68]. Nevertheless, Tregs were not detected in the hippocampus in the present study. Therefore, the divergent changes of cytokines in the present study are probably attributed to the anatomic structure of the blood–brain barrier.

The blood–brain barrier provides primary protection by limiting the solutes in the circulating blood to the extracellular fluid of the central nervous system. In the present study, the increased signals of low-molecule dextran and reduced presence of tight junction proteins in aged mice following the surgery confirm the blood–brain barrier disruption. Treatment of the CD25 antibody restored the protein presence of junction proteins, leading to reduced TNF-α and CXCL1 levels in the hippocampus. The reduced levels of inflammatory factors in mice hippocampi, especially TNF-α, reinforce the crucial role of TNF-α and its pertinent receptors in aged Tregs in POCD [69, 70]. It further demonstrates that Tregs blockade results in cognition-protective effects through modulating TNF-α levels and BBB structures in the hippocampus in aged mice challenged with surgery. Thus, screening for protective substances produced by the blockade of the CD25 molecule and dissecting the mechanisms underlying the upregulation of endothelial junction proteins would enhance our understanding of the connection between immune status in circulating blood and the central nervous system.

In addition, CD4+CD25-Foxp3+ cells, another suppressive group of lymphocytes [71], were also accumulated in aged mice. The involvement of CD4+CD25-Foxp3+ cells in surgery-associated cognitive dysfunction has not been reported and deserves further investigation.

In the present study, transfusion with young Tregs improved swimming velocity in aged mice. The heterochronic parabiosis of young cells in aged mice has been extensively studied [72, 73]. In a mouse model of human Duchenne muscular dystrophy, muscle injury and inflammation is mitigated by Tregs expansion, but exacerbated by Treg depletion [74]. The graft Tregs-exerted protection is probably attributed to tissue repair by acting on parenchymal cells directly [73, 75–77].

In conclusion, surgery-associated cognitive decline in aged mice is attributed to Tregs dysfunction. Blocking the CD25 molecule protects the blood–brain barrier, downregulates inflammation in the hippocampus, and restores cognitive function in aged mice. The results of the present study provide a therapeutic strategy for postoperative cognitive dysfunction in the geriatric population.

## Supplementary Information

**Additional file 1: Figure S1.** Four shots of ATRA significantly increased counts of splenic CD4+CD25+Foxp3+ Tregs but had lethal effects on aged ones. **Figure S2.** Verification of isolated Tregs by the Regulatory T Cell Isolation Kit. **Figure S3.** Tregs from Foxp3^YFP^ mice were injected into aged mice via tail vein subjected to the surgery. **Figure S4.** Swimming velocity documented in the Morris maze test. **Figure S5.** Changes of LAG-3 (A) and HELIOS (B) proteins in Tregs under basal and stimulated conditions in flow cytometry. **Figure S6.** Bodyweight of mice with Tregs ablation. **Figure S7.** Cytokines expressions in plasma and hippocampus of mice with Tregs ablation.

## Data Availability

The RNA-sequencing data produced in the present study were uploaded to the NCBI–GEO database. https://www.ncbi.nlm.nih.gov/geo/query/acc.cgi?acc=GSE208231.
