# Supplementary material for: Tregs dysfunction aggravates postoperative cognitive impairment in aged mice
Source: J Neuroinflammation. 2023 Mar 17;20:75. doi: 10.1186/s12974-023-02760-7 (PMC10022212; doi:10.1186/s12974-023-02760-7)
Supplement: Supplementary file 1 — Additional file 1: Figure S1. Four shots of ATRA significantly increased counts of splenic CD4+CD25+Foxp3+ Tregs but had lethal effects on aged ones. Figure S2. Verification of isolated Tregs by the Regulatory T Cell Isolation Kit. Figure S3. Tregs from Foxp3YFP mice were injected into aged mice via tail vein subjected to the surgery. Figure S4. Swimming velocity documented in the Morris maze test. Figure S5. Changes of LAG-3 (A) and HELIOS (B) proteins in Tregs under basal and stimulated conditions in flow cytometry. Figure S6. Bodyweight of mice with Tregs ablation. Figure S7. Cytokines expressions in plasma and hippocampus of mice with Tregs ablation. [file 12974_2023_2760_MOESM1_ESM.docx]

Tregs dysfunction aggravates postoperative cognitive impairment in aged mice

Yile Zhou^1*^, Huihui Ju^1*^, Yan Hu^1*^, Tingting Li^2 3^, Zhouyi Chen^1^, Yuan Si^4^, Xia Sun^5^, Yi Shi^2 3^, Hao Fang^1 4^

1. Department of Anesthesiology, Zhongshan Hospital, Fudan University, Shanghai, China

2. Institute of Clinical Science, Zhongshan Hospital, Fudan University, Shanghai, China

3. Shanghai Key Laboratory of Organ Transplantation, Zhongshan Hospital, Fudan University, Shanghai, China

4. Department of Anesthesiology, Minhang Branch, Zhongshan Hospital, Fudan University, Shanghai, China

5. Department of Anesthesiology, Shanghai Cancer Center, Fudan University, Shanghai, China

These authors contributed to the study equally.

Corresponding Author

Professor Hao Fang

Department of Anesthesiology, Zhongshan Hospital, Fudan University, Shanghai, China

E-mail: [drfanghao@163.com](mailto:drfanghao@163.com)

Dr. Yi Shi

Institute of Clinical Science, Zhongshan Hospital, Fudan University, Shanghai, China

Shanghai Key Laboratory of Organ Transplantation, Zhongshan Hospital, Fudan University, Shanghai, China

E-mail: [shi.yi@zs-hospital.sh.cn](mailto:shi.yi@zs-hospital.sh.cn)

ORCID: 0000-0003-3005-9655

Dr. Xia Sun

Department of Anesthesiology, Shanghai Cancer Center, Fudan University, Shanghai, China

E-mail: [18017312998@163.com](mailto:18017312998@163.com)

Additional Figure S1


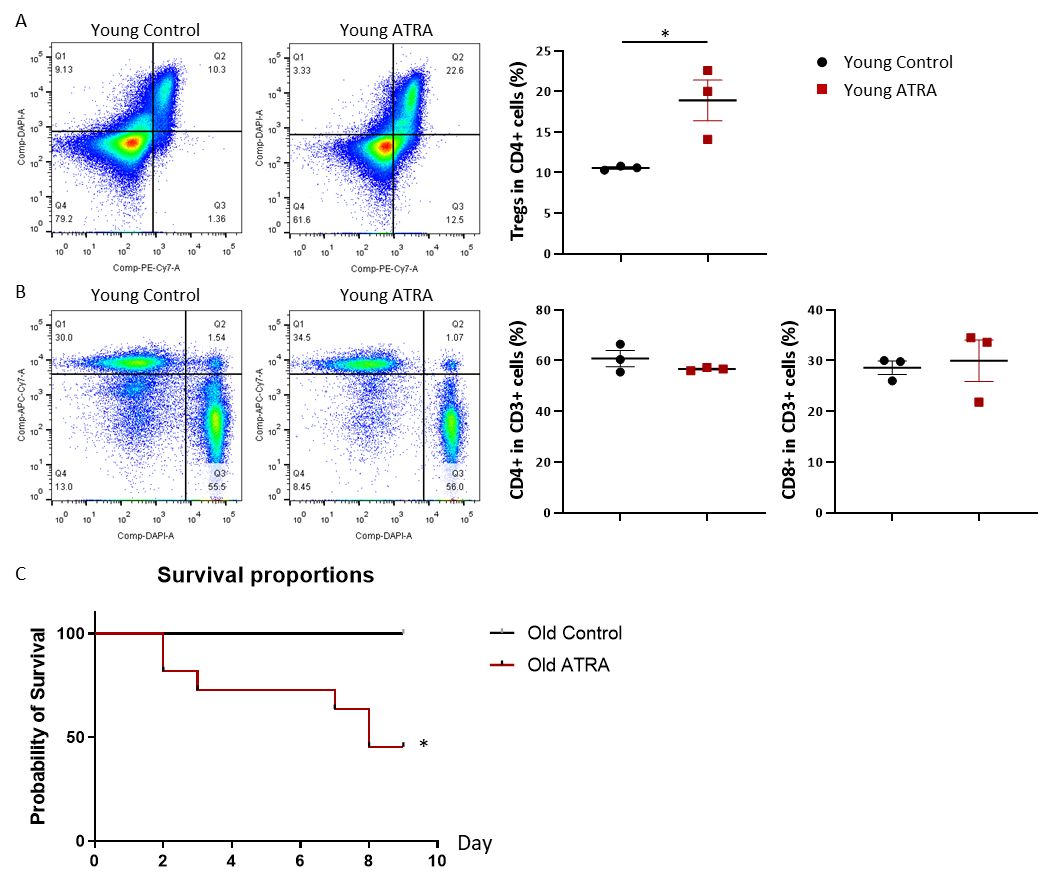


Additional Figure S1. Four shots of ATRA significantly increased counts of splenic CD4+CD25+Foxp3+ Tregs but had lethal effects on aged ones. A. ATRA increased peripheral Tregs in young mice. DAPI: CD25, PE-Cy7: Foxp3. B. ATRA failed to increase CD4 or CD8 cells in young mice. APC-Cy7: CD8, DAPI: CD4. C. ATRA had lethal effects on aged mice. About 50% of mice (n=9) were dead after four shots of ATRA (4 mg/kg), and the remaining mice did not survive after the surgery

Additional Figure S2


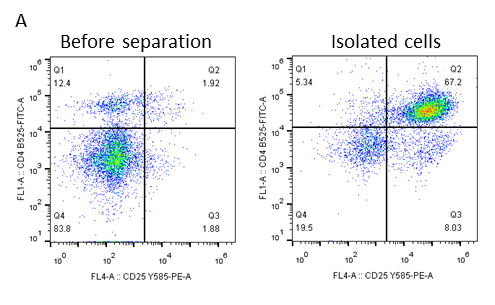


Additional Figure S2. Verification of isolated Tregs by the Regulatory T Cell Isolation Kit.

Additional Figure S3


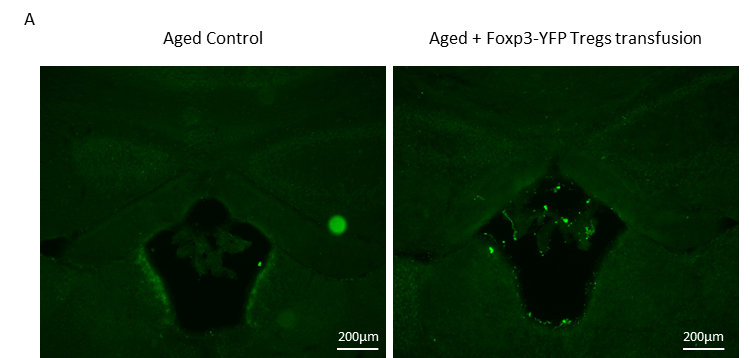


Additional Figure S3. Tregs from Foxp3^YFP^ mice were injected into aged mice via tail vein subjected to the surgery. The YFP signal was detected in the choroid plexus, but not the hippocampi of the mice. Green: YFP. Magnification, 100×.

Additional Figure S4


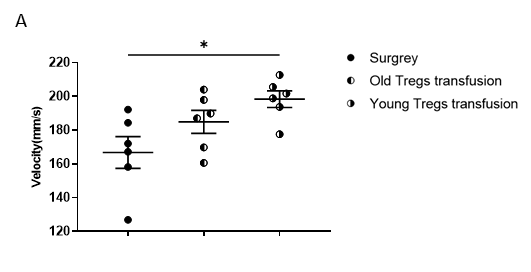


Additional Figure S4. Swimming velocity documented in the Morris maze test. P<0.05 n=6

Additional Figure S5


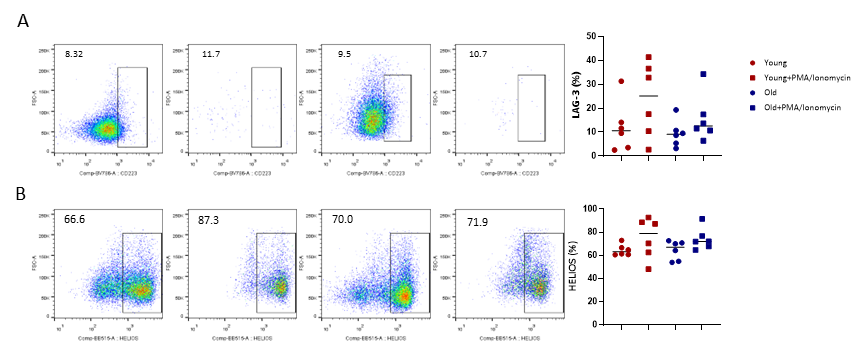


Additional Figure S5. Changes of LAG-3 (A) and HELIOS (B) proteins in Tregs under basal and stimulated conditions in flow cytometry.

Additional Figure S6


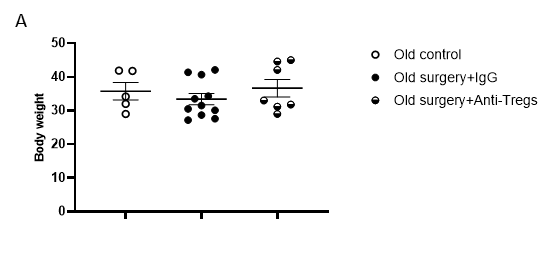


Additional Figure S6. Bodyweight of mice with Tregs ablation. n=5-11


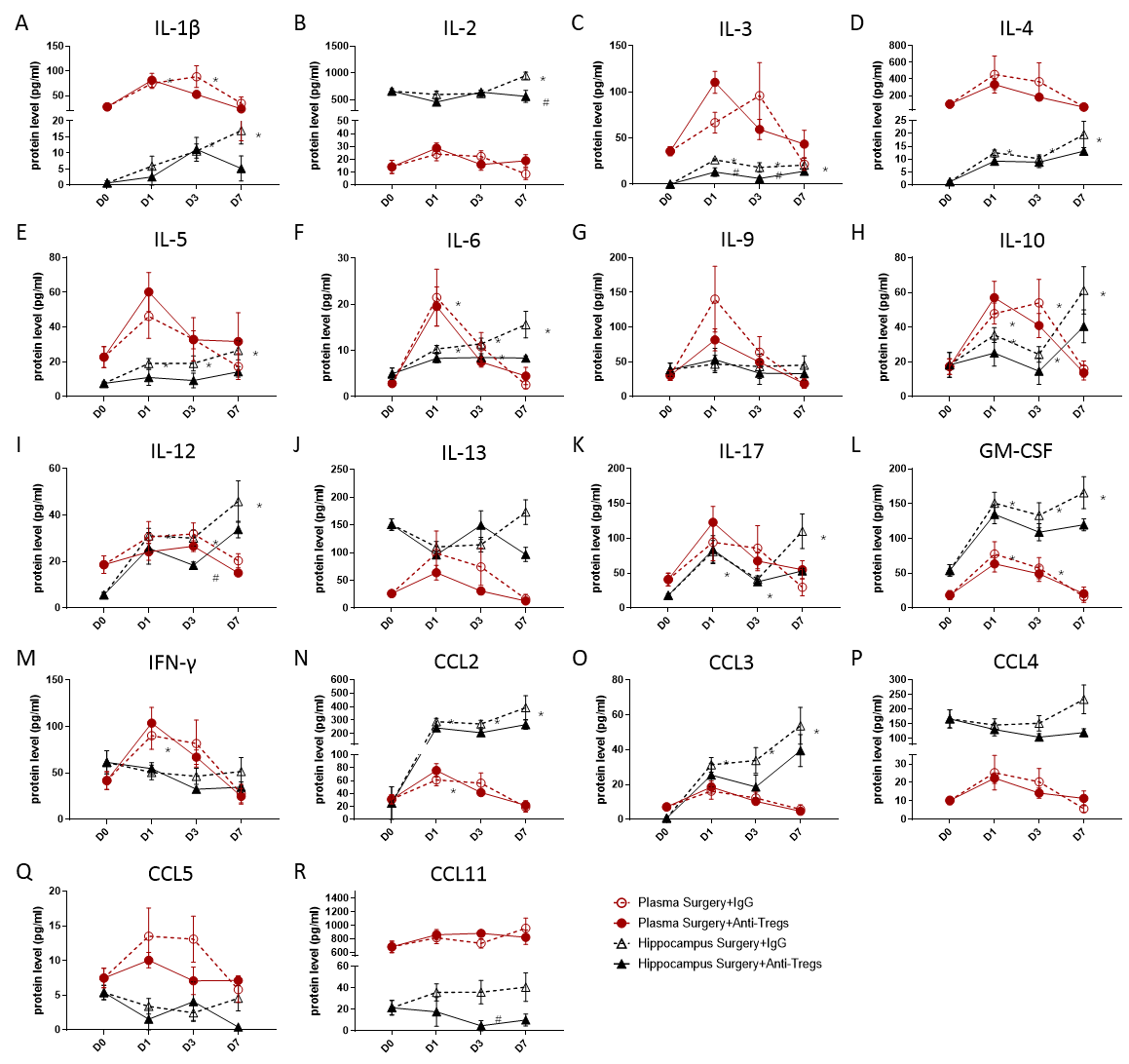
Additional Figure S7

Additional Figure S7. Cytokines expressions in plasma and hippocampus of mice with Tregs ablation. * P<0.05 vs. D0, # P<0.05 vs. Surgery+IgG on the same day. n=6
